# Determinants of time to positivity in bloodstream infections: an analysis of a population-based cohort in Queensland, Australia

**DOI:** 10.1007/s10096-025-05096-7

**Published:** 2025-03-08

**Authors:** Felicity Edwards, Michael Waller, Kevin B. Laupland

**Affiliations:** 1 Queensland University of Technology (QUT), Brisbane, QLD Australia; 2 School of Public Health, University of Queensland, Brisbane, QLD Australia; 3 Department of Intensive Care Services, Royal Brisbane and Women’S Hospital, Level 3 Ned Hanlon Building, Butterfield Street, Brisbane, QLD 4029 Australia

**Keywords:** Time to positivity, TTP, Bloodstream infection, BSI, Bacterial load, Population based

## Abstract

**Purpose:**

Time to Positivity (TTP) measures the interval from incubation to bacterial growth detection in blood cultures. Although shorter TTP is associated with higher mortality, factors associated with TTP remain uncertain.

**Methods:**

A retrospective cohort study was conducted among Queensland residents with positive blood cultures between 2000–2019. Incident bloodstream infections (BSIs) were identified using Pathology Queensland data, with demographic, clinical, and outcome data linked to state-wide databases.

**Results:**

The study analysed 84,341 patients with monomicrobial BSI with a median patient age of 65.6 years (IQR 45.4–78.1), and most infections being community-associated (77.0%). Age showed a non-linear relationship with TTP, and male sex was linked with slightly higher TTP (Incidence Rate Ratio (IRR) 1.01; 95% Confidence Intervals (CI) 1.00–1.02; *p* = 0.011), reflecting a small but measurable association. Liver disease and malignancy were associated with lower TTP (IRR 0.93; 95% CI 0.91–0.95; *p* < 0.0001 and IRR 0.95; 95% CI 0.94–0.97; *p* < 0.0001 respectively), whilst diabetes showed no significant difference (IRR 1.01; 95% CI 1.00–1.02; *p* = 0.0840). Hospital onset infections exhibited longer TTPs (IRR 1.09; 95% CI 1.08–1.10; *p* < 0.0001).

**Conclusions:**

There are several host characteristics associated with TTP that may in part explain the complex relationship between this variable and mortality. Beyond microbiological factors such as isolate type, TTP is also influenced by clinical variables including patient demographics and infection characteristics highlighting its potential as a prognostic marker. Further evaluation is needed to clarify its role in predicting patient outcomes and guiding tailored treatment strategies.

## Introduction

Time to Positivity (TTP) refers to the interval from the start of bacterial incubation to the detection of bacterial growth in a blood culture [1–4]. TTP is highly variable among different bacterial species due to their individual characteristics. However, when considering specific species, TTP is thought to reflect the bacterial load at the time of collection, as shorter TTPs are generally associated with higher initial bacterial doses [4]. TTP is increasingly recognised as a valuable clinical tool for assessing treatment responses and prognoses, with shorter TTP being associated with higher fatality rates and other adverse outcomes [1, 5].

Although TTP has been studied primarily as a predictor of mortality, its broader clinical implications remain unclear [1, 2, 6]. Previous research is often limited by a narrow focus on death, small sample size, short study durations and restricted geographical coverage [1, 2, 6]. To address these gaps, this study investigates how clinical factors, such as comorbidities, infection source and specific organisms influence TTP in blood cultures. Recent work by Laupland et al. demonstrated significant association between shorter TTP and increased mortality risk in bloodstream infections [7]. The present study builds upon this research by further examining the clinical and microbiological determinants influencing TPP, which may help contextualised its prognostic value beyond its link to mortality.

## Methods

### Study design

A retrospective cohort design was conducted to study all Queensland, Australia residents (population approximately 5 million) with mono-microbial bloodstream infections (BSI) identified within the publicly funded healthcare system between January 1, 2000, and December 31, 2019. Ethical approval was granted by the Royal Brisbane and Women’s Hospital Human Research Ethics Committee with a waiver of individual consent (LNR/2020/QRBW/62494).

### Participants

The inclusion and exclusion criteria for this study were identical to those used in Laupland et al. [7], ensuring consistency with the original cohort selection. Specifically, this study included only incident monomicrobial BSI, as defined in the prior study [7]. Patients with polymicrobial infections were excluded and for those with multiple BSI during the surveillance period, a single incident episode was randomly selected for analysis [7].

### Procedures

Positive blood cultures were identified through Pathology Queensland, which services both community and institutional settings across the state. For most of the study period, the BACT/Alert 3D system (bioMérieux, Inc, Durham, NC) was used for culturing, with the BACT/ALERT VIRTUO system (bioMériux, Inc, Durham, NC) implemented in 2018 for the Greater Brisbane area and some rural sites.

Validated algorithms were applied to identify and classify incident infections as previously described [8]. Incident BSI was defined as the initial isolation of a species from a patient, with any repeat isolation of the same species within a 30-day period classified as the same episode. To gather demographic, clinical and outcome information, Incident BSI cases were linked with state-wide hospital admissions and vital statistics databases [9]. International Classification of Diseases-10 Australian Modification (ICD-10-AM) discharge codes were collected for all admissions within two years prior to and one year post incident BSI. Charlson comorbidity scores were calculated as per Quan et al., and infection focus was determined using primary discharge ICD-10-AM codes [10]. Organisms were categorised into twenty pre-specified species based on relative frequency, microbiological characteristics, and previous studies [2].

Time to positivity was originally recorded in Pathology Queensland’s database as free text entries and subsequently converted to numerical format for analysis. For consistency, TTP was rounded down to the nearest whole hour, as some values included decimal points (e.g. 1.2, 1.25, or 1.9), and it was unclear whether these decimals represented fractions of an hour or minutes.

### Analysis

Demographic and clinical characteristics were stratified by clinically relevant time intervals based on expert clinical reasoning to ensure the categories reflected meaningful differences in infection dynamics, the stratification was then summarised using descriptive statistics. Non-parametric trend analysis was performed using Cuzick’s test for trend to identify patterns across ordered groups and group comparisons for continuous variables were performed using the Kruskal–Wallis test [11, 12].

Time to positivity was modelled as a continuous outcome using a multivariable Inverse Gaussian Generalised Linear Model (GLM) with a log link function. This approach was chosen due to the right-skewed distribution of TTP [13, 14]. Although TTP is recorded in hours, it represents a continuous measurement rather than a discrete count outcome. Prior to selecting this model, alternative approaches including linear regression and various transformations (Log, Square Root and Box Cox) were tested, but model diagnostics indicated that the Inverse Gaussian GLM best captured the underlying data distribution [13].

Backward elimination was used to refine the model, systematically removing non-significant variables based on *p*-value thresholds and clinical relevance [15]. Model fit was evaluated using the Pearson and Deviance measures, along with the Bayesian Information Criterion (BIC), with lower values indicating better model fit [15–18]. The Incidence Rate Ratios (IRR) from this model represent the relative change in expected TTP for a given predictor variable, with values < 1 indicating a shorter TTP and values > 1 indicating a longer TTP. For example, an IRR of 0.93 for liver disease suggests that patients with liver disease have a 7% shorter TTP compared to those without liver disease. This interpretation is consistent across all modelled variables.

**Fig. 1:**
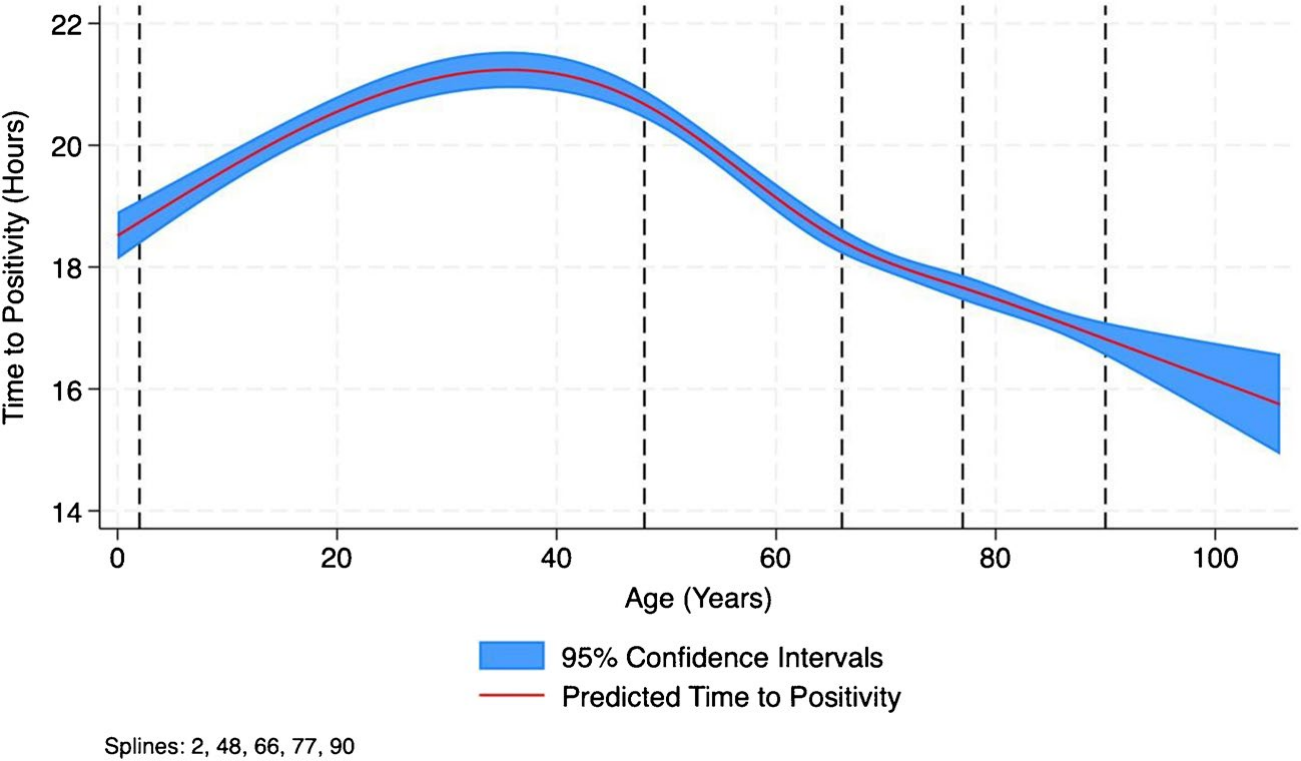
Age vs time to positivity model using restricted cubic splines. The red line represents the predicted time to positivity (TTP) across age, modelled using restricted cubic splines. The blue shaded area represents the 95% confidence intervals for the predictions, which widen at extreme ages (younger than 2 and older than 90 years) due to lower data density and higher model uncertainty. The curvature reflects how the relationship between age and TTP varies across the lifespan

To capture the non-linear relationship between age and TTP, restricted cubic splines were used to provide a flexible modelling approach that did not assume a specific parametric form [19, 20]. The spline adjusted IRRs reflect relative changes in TTP as specific age points, rather than assuming a uniform effect across all ages. Figure 1 visually illustrates these relationships with 95% confidence intervals.

**Fig. 2:**
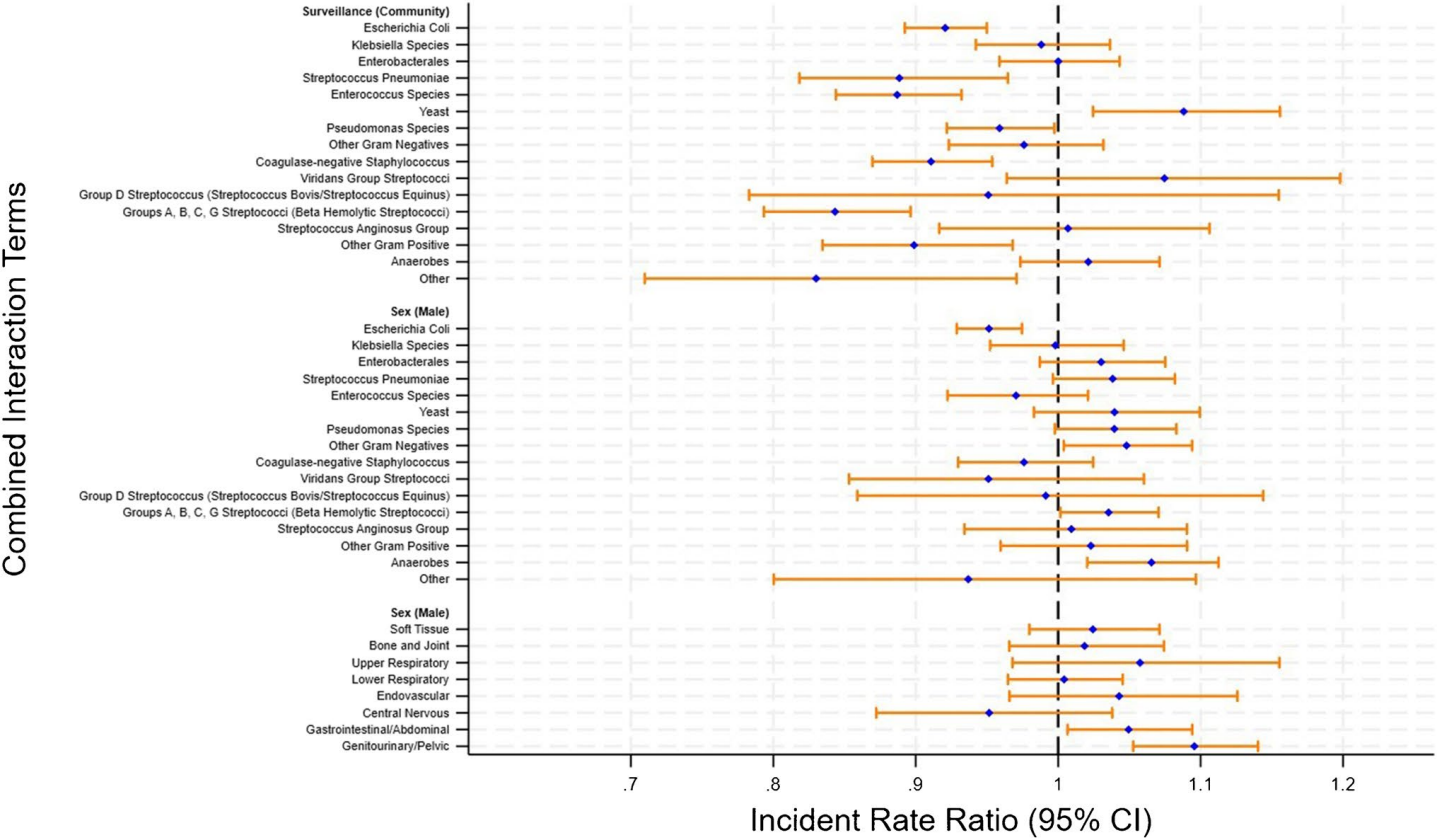
Forest plot illustrating interactions: (1) isolate group with community-onset vs hospital infections (top), (2) isolate group with sex (middle), and (3) focus of infection by sex (bottom). Reference groups: 1) hospital infections, 2) isolate—*Staphylococcus aureus*, 3) sex—female

Interaction terms were included to account for potential effect modification between clinical and microbiological factors and their influence on TTP (Fig. 2). Specifically, interactions between isolate groups and infection origin (community vs. hospital onset), sex, and focus of infection were considered. These terms were selected based on prior evidence and biological plausibility, suggesting that the relationship between TTP and patient outcomes may vary depending on isolate characteristics, the clinical context of infection, and patient demographics [4, 21, 22]. The interaction terms aimed to provide a more nuanced understanding of how these variables jointly influence TTP, ensuring accurate representation of their relationships and appropriate adjustment for potential confounders [23].

All statistical analyses were conducted using Stata 18.0 (StataCorp, College Station, Texas) with statistical significance set at *p* < 0.05.

## Results

### Population characteristics

A total of 84,341 incident episodes were included in the analysis, with males accounting for 55.7% of the cohort (46,844/84,132). The sex distribution showed a significant trend (Cuzicks test for trend *p* < 0.0001). The median age was 65.6 years (Interquartile range (IQR); 45.4–78.1) with significant variation across time to positivity groups (Cuzicks test for trend *p* < 0.0001). Almost 60% of infections had a time to positivity within the first sixteen hours of collection (49,988/84,132; 59.4%). Most infections were community-associated (64,823/84,132; 77.0%) and comorbidities were common, with diabetes mellitus, malignancy, renal disease the most prevalent as shown in Table 1.

**Table 1:** Clinical characteristics stratified by time to positivity in hours

Factor	Total *n* = *84,132*	0 to < 8 h *n* = *10,298*	8 to < 16 h *n* = *39,690*	16 to < 24 h *n* = *16,486*	> 24h *n* = *17,658*	*p*-value
Median age (IQR)	65.6 (45.4–78.1)	69.4 (52.5–80.1)	66.7 (46.9–78.8)	64.2 (42.2–77.1)	61.8 (40.9–75.7)	0.0001
Male (%)	46,844 (55.7)	5,447 (52.9)	21,433 (54.0)	9,695 (58.8)	10,269 (58.2)	0.0001
Onset (%)Hospital-onset infection	19,309 (23.0)					0.0001
Community-onset infection	64,823 (77.0)	1,552 (15.1) 8,746 (84.9)	7,931 (20.0) 31,759 (80.0)	4,492 (27.3) 11,994 (72.7)	5,334 (30.2) 12,324 (69.8)	
Day 30 mortality (%)	10,682 (12.7)	1,487 (14.4)	4,850 (12.2)	2,065 (12.5)	2,280 (12.9)	0.1540
Comorbidities (%)						
Myocardial infarction	7,371 (8.8)	948 (9.2)	3,452 (8.7)	1,498 (9.1)	1,473 (8.3)	0.0737
Congestive heart failure	13,197 (15.7)	1,761 (17.1)	6,375 (16.1)	2,451 (14.9)	2,610 (14.8)	0.0001
Peripheral vascular disease	5,032 (6.0)	629 (6.1)	2,220 (5.6)	1,032 (6.3)	1,151 (6.5)	0.0007
Cardiovascular disease	5,774 (6.9)	747 (7.3)	2,629 (6.6)	1,117 (6.8)	1,281 (7.3)	0.2334
Dementia	4,679 (5.6)	596 (5.8)	2,321 (5.9)	895 (5.4)	867 (4.9)	0.0001
Pulmonary disease	9,841 (11.7)	1,218 (11.8)	4,497 (11.3)	1,976 (12.0)	2,150 (12.2)	0.0226
Rheumatic	1,466 (1.7)	179 (1.7)	655 (1.7)	301 (1.8)	331 (1.9)	0.0939
Peptic ulcer disease	1,668 (2.0)	196 (1.9)	715 (1.8)	328 (2.0)	429 (2.4)	0.0001
Liver disease	6,785 (8.1)	855 (8.3)	3,310 (8.3)	1,244 (7.6)	1,376 (7.8)	0.0063
Diabetes mellitus	20,385 (24.2)	2,609 (25.3)	9,765 (24.6)	3,954 (24.0)	4,057 (23.0)	0.0001
Plegia	4,019 (4.8)	483 (4.7)	1,770 (4.5)	863 (5.2)	903 (5.1)	0.0006
Renal disease	14,583 (17.3)	1,892 (18.3)	7,096 (17.9)	2,805 (17.0)	2,790 (15.8)	0.0001
Malignancy	15,321 (18.2)	1,692 (16.4)	7,224 (18.2)	3,106 (18.8)	3,299 (18.7)	0.0001
HIV	172 (< 1.0)	12 (< 1.0)	79 (< 1.0)	33 (< 1.0)	48 (< 1.0)	0.0103
Focus of infection (%)						0.0006
No focus/primary	41,419 (49.2)	4,982 (48.4)	19,676 (49.6)	8,292 (50.3)	8,469 (48.0)	
Soft tissue	5,422 (6.4)	726 (7.1)	2,417 (6.1)	1,168 (7.1)	1,111 (6.3)	
Bone and joint	3,359 (4.0)	299 (2.9)	1,507 (3.8)	886 (5.4)	667 (3.8)	
Upper respiratory	992 (1.2)	42 (< 1.0)	340 (< 1.0)	210 (1.3)	400 (2.3)	
Lower respiratory	7,706 (9.2)	862 (8.4)	3,516 (8.9)	1,596 (9.7)	1,732 (9.8)	
Endovascular	1,945 (2.3)	337 (3.3)	998 (2.5)	315 (1.9)	295 (1.7)	
Central nervous	926 (1.1)	122 (1.2)	430 (1.1)	202 (1.2)	172 (< 1.0)	
Gastrointestinal/abdominal	9,667 (11.5)	1,145 (11.2)	4,295 (10.8)	1,601 (9.7)	2,626 (14.9)	
Genitourinary/pelvic	12,696 (15.1)	1,783 (17.3)	6,511 (16.4)	2,216 (13.4)	2,186 (12.4)	
Organism/group (%)						0.0001
*Staphylococcus aureus*	15,582 (18.5)	1,595 (15.5)	6,909 (17.4)	4,370 (26.5)	2,708 (15.3)	
*Escherichia coli*	22,960 (27.3)	4,046 (39.3)	13,944 (35.1)	2,726 (16.5)	2,244 (12.7)	
*Klebsiella* species	5,620 (6.7)	907 (8.8)	3,272 (8.2)	733 (4.5)	690 (3.9)	
Other Enterobacterales*	6,466 (7.7)	750 (7.3)	3,505 (8.8)	1,303 (7.9)	908 (5.1)	
*Streptococcus pneumoniae*	3,903 (4.6)	541 (5.3)	2,714 (6.8)	387 (2.4)	261 (1.5)	
*Entrococcus* species	2,688 (3.2)	313 (3.0)	1,492 (3.8)	612 (3.7)	271 (1.5)	
Yeast	1,480 (1.8)	7 (< 1.0)	75 (< 1.0)	163 (< 1.0)	1,235 (7.0)	
*Pseudomonas* species	4,088 (4.9)	167 (1.6)	1,097 (2.8)	1,980 (12.0)	844 (4.8)	
Other Gram-negatives	5,477 (6.5)	315 (3.0)	1,322 (3.3)	1,352 (8.2)	2,488 (14.1)	
Coagulase-negative staphylococci	2,390 (2.8)	34 (< 1.0)	364 (< 1.0)	1,073 (6.5)	919 (5.2)	
Viridans group streptococci	818 (< 1.0)	39 (< 1.0)	375 (< 1.0)	256 (1.6)	148 (< 1.0)	
Group D streptococci	335 (< 1.0)	48 (< 1.0)	221 (< 1.0)	34 (< 1.0)	32 (< 1.0)	
Groups A, B, C/G streptococci	5,819 (6.9)	1,382 (13.4)	3,795 (9.6)	380 (2.3)	262 (1.5)	
*Streptococcus anginosus* group	1,024 (1.2)	14 (< 1.0)	61 (< 1.0)	274 (1.7)	675 (3.8)	
Other Gram-positive	1,811 (2.2)	60 (< 1.0)	231 (< 1.0)	389 (2.4)	1,131 (6.4)	
Anaerobes	3,516 (4.2)	77 (< 1.0)	305 (< 1.0)	445 (2.7)	2,689 (15.2)	
Other	173 (< 1.0)	3 (< 1.0)	8 (< 1.0)	9 (< 1.0)	153 (< 1.0)	

^*^Excluding *E.coli* and *Klebsiella* species which are reported separately

In multivariate analysis, age demonstrated a non-linear relationship with TTP. The TTP increased during early adulthood, peaking around 40 years of age, and subsequently declining progressively with advancing age. This decline became particularly steep and pronounced beyond 70 years of age, as shown in Fig. 1 and Table 2. Male sex was associated with a slightly higher TTP compared to females (IRR) 1.01; 95% Confidence Interval (CI) 1.00–1.02; *p* = 0.011) and community onset infections were associated with a lower TTP compared to hospital onset infections (IRR 0.92; 95% CI 0.91–0.93; *p* < 0.0001). Comorbid conditions had varied effects on TTP with only liver disease (IRR 0.93; 95% CI 0.91–0.95; *p* < 0.0001) and malignancy (IRR 0.95; 95% CI 0.94–0.97; *p* < 0.0001) showing a significant reduction in TTP. Diabetes did not have a significant relationship with TPP (IRR 1.01; 95% CI 1.00–1.02; *p* = 0.0840).

**Table 2:** Inverse Gaussian generalised linear model of risk factors associated with time to positivity (TTP)

Variable	Incidence Rate Ratio	95% Confidence Interval	*p*-value
Sex			
Female	ref	-	-
Male	1.01	1.00—1.02	0.0110
Age*			
2 years	ref	-	-
around 48 years	1.00	1.00—1.00	< 0.0001
around 66 years	0.99	0.99—1.00	< 0.0001
around 77 years	1.04	1.02—1.07	< 0.0001
around 90 years	0.93	0.86—1.00	0.0640
Surveillance			
Hospital	ref	-	-
Community	0.92	0.91—0.93	< 0.0001
Comorbidities			
Liver disease	0.93	0.91—0.95	< 0.0001
Malignancy	0.95	0.94—0.97	< 0.0001
Diabetes	1.01	1.00—1.02	0.0840
Focus of infection			
No focus/primary	ref	-	-
Soft tissue	1.05	1.03—1.07	< 0.0001
Bone and joint	1.05	1.03—1.08	< 0.0001
Upper respiratory	1.12	1.07—1.17	< 0.0001
Lower respiratory	1.06	1.04—1.08	< 0.0001
Endovascular	0.82	0.79—0.85	< 0.0001
Central nervous	0.85	0.82—0.88	< 0.0001
Gastrointestinal/abdominal	1.05	1.03—1.06	< 0.0001
Genitourinary/pelvic	1.12	1.10—1.14	< 0.0001
Organism/group			
*Staphylococcus aureus*	ref	-	-
*Escherichia coli*	0.78	0.76—0.79	< 0.0001
*Klebsiella* species	0.85	0.83—0.87	< 0.0001
Enterobacterales	0.94	0.92—0.96	< 0.0001
*Streptococcus pneumoniae*	0.73	0.71—0.75	< 0.0001
*Entrococcus* species	0.87	0.85—0.90	< 0.0001
Yeast	2.56	2.47—2.62	< 0.0001
*Pseudomonas* species	1.18	1.15—1.20	< 0.0001
Other Gram-negatives	1.69	1.65—1.73	< 0.0001
Coagulase-negative staphylococci	1.42	1.39—1.46	< 0.0001
Viridans group streptococci	1.19	1.13—1.25	< 0.0001
Group D streptococcus	0.81	0.75—0.87	< 0.0001
Groups A, B, C/G streptococci	0.64	0.63—0.65	< 0.0001
*Streptococcus anginosus* group	1.97	1.90—2.05	< 0.0001
Other Gram-positive	2.10	2.03—2.16	< 0.0001
Anaerobes	2.44	2.38—2.50	< 0.0001
Other	3.63	3.35—3.92	< 0.0001

^*^Each spline term (at ages 2, 48, 66, 77, and 90 years) reflects the change in TTP, expressed as a ratio, relative to the reference point (age 2). A ratio > 1 indicates a percentage increase in TTP, while a ratio < 1 indicates a percentage decrease. These ratios reflect changes specific to the area around the corresponding ages (knots) shown in Fig. 1 and are part of the smooth continuous relationship modelled by splines. For example, an incidence rate ratio of 1.04 at around 77 years suggest a 4% increase in TTP compared to age 2 in this part of the curve. These ratios should be interpreted in the context of the overall non-linear relationship shown in Fig. 1. It is important to note that the results in this table present relative changes at specific points (splines), while Fig. 1 visualises the overall predicted trend in TTP across the entire age range. As such, the figure may show higher TTP values at certain ages (e.g., 48 years) due to the cumulative, non-linear effects of all spline terms, even if the table indicates no significant relative change at these points

### Infection site characteristics

Bivariate analysis showed genitourinary/pelvic focus was associated with shorter TTP, while lower respiratory tract focus exhibited longer TTP. In multivariate analysis, these trends reversed with genitourinary/pelvic and lower respiratory associated with longer TTP (IRR 1.12; 95% CI 1.10–1.14; *p* < 0.0001 and IRR 1.06; 95% CI 1.04–1.08; *p* < 0.0001 respectively). By contrast, only infections classified as endovascular and central nervous displayed shorter TTP (IRR 0.82; 95% CI 0.79–0.85; *p* < 0.0001 and IRR 0.85; 95% CI 0.82–0.88; *p* < 0.0001).

### Microbiological characteristics

Overall, *Escherichia coli* and *Staphylococcus aureus* were the most frequently isolated organisms accounting for almost half of cases collectively (38,542/84,132; 45.8%). In the bivariate analysis presented in Table 1, significant variation in TTP was observed among different isolates, for example *E. coli* and groups A, B, C/G streptococci exhibited shorter TTP, while coagulase-negative staphylococci and anaerobes showed notably longer TTP. In multivariate analysis, isolate type remained a strong determinant of TTP. Groups A, B, C/G streptococci continued to exhibit shorter TTP (IRR 0.64; 95% CI 0.63–0.65;* p* < 0.0001) as did *E. coli (*IRR 0.78; 95% CI 0.76–0.79; *p* < 0.0001). Similarly, *Streptococcus pneumoniae,* and Group D streptococcus were associated with reduced TTPs (IRR 0.73; 95% CI 0.71–0.74; *p* < 0.0001 and IRR 0.81; 95% CI 0.75–0.87; *p* < 0.0001 respectively). In contrast, Yeast and Anaerobes were associated with the longest TTPs (IRR 2.55; 95% CI 2.47–2.62;* p* < 0.0001 and IRR 2.44; 95% CI 2.38–2.50; *p* < 0.0001).

### Interactions

The forest plots in Fig. 2 show the interaction effect between isolate type and key clinical factors such as community onset versus hospital onset infection, sex and infection site. The interaction between infection onset and isolate type showed that Group A, B, C/G streptococci (IRR 0.84; 95% CI 0.79–0.90; *p* < 0.0001), *E.coli* (IRR 0.92; 95% CI 0.89–0.95; *p* < 0.0001), *Streptococcus pneumoniae* (IRR 0.89; 95% CI 0.82–0.96; *p* = 0.0050) and *Enterococcus* species (IRR 0.87; 95% CI 0.84–0.93; *p* < 0.0001) exhibited shorter TTP for community onset infections compared to hospital onset infections, which are consistent trends with the multivariate results. Additionally, community onset infection for yeast demonstrated a longer TTP (IRR 1.08; 95% CI 1.02–1.16; *p* = 0.0060) than hospital onsets. Notably, *Pseudomonas* species (IRR: 0.96; 95% CI 0.92–0.99; *p* = 0.0370), coagulase negative staphylococci (IRR; 0.91; 95% CI 0.87–0.95; *p* < 0.0001) and other gram positive (IRR 0.90; 95% CI; 0.83–0.97; *p* = 0.0500) all displayed significantly shorter TTP for community onset infections compared to hospital onsets.

The interaction between sex and isolate group showed that *E. coli* maintained a shorter TTP in males compared to females (IRR 0.95; 95% CI 0.93–0.97; *p* < 0.0001) highlighting a distinct isolate specific interaction effect. This finding contrasts with the overall trend observed in the population, where males generally exhibited a slightly longer TTP compared to females. By contrast, Group A, B, C/G streptococci exhibited longer TTP in males (IRR 1.03; 95% CI 1.00–1.07; *p* = 0.0400). For the interaction between infection site and sex, males showed longer TTP for gastrointestinal/abdominal infections (IRR 1.05; 95% CI 1.01–1.09; *p* = 0.0230) and genitourinary/pelvic infections (IRR 1.10; 95% CI 1.05–1.14; *p* < 0.0001). These findings highlight notable differences in TTP based on infection site and sex, emphasising the distinct roles these factors play in determining TTP.

## Discussion

In this study, we investigated the association between TTP and clinical factors, including comorbidities, focus of infection, and isolate type, in a large population-based cohort of bloodstream infections in Queensland Australia. While hospital onset infections were associated with longer TTPs, comorbidities such as liver disease and malignancy were linked to shorter TTP. Variations in TTP across infection sites and bacterial isolates, reflecting the role of infection focus and isolate type in shaping bacterial growth dynamics. By examining a wide range of clinical variabes, this study significantly advances understanding of the factors influencing TTP.

This study advances the understanding of TTP by addressing gaps in prior research. Unlike previous research that focused on mortality as the primary outcome and were constrained by small sample sizes, limited study durations and narrow geographical scope [5, 21, 22, 24–30], our research includes a large population based cohort of 84, 341 episodes over two decades in Queensland Australia. This expansive dataset enables a detailed examination of TTP in relation to a broad spectrum of clinical factors, beyond mortality outcomes. By incorporating a diverse range of clinical variables, the study enhances the clinical relevance of TTP by providing a more nuanced understanding of its variability and associations across different patient contexts. Our findings suggest that TTP can be more than an indicator of bacterial load; when integrated with other variables, it can support the development of more comprehensive prognostic models [31, 32]. These models could enable clinicians to predict patient outomes more accurately and tailor treatment strategies accordingly,

Our findings reveal notable associations between comorbidities and TTP. Liver disease and malignancy were linked to shorter TTPs, which may reflect immune dysfunction, altered microbial dynamics or higher initial bacterial loads [28]. Similar trends have been observed in studies examining liver disease, where compromised hepatic function was associated with increased susceptibiltiy to invasive infections and altered pathogen clearance [28]. Malignancy, on the other hand, is often linked to immunosuppressive states, either due to the disease itself or its treatments, potentially facilitating rapid bacterial proliferation [28]. Contrary to our expectations, diabetes was not significantly associated with TTP. We hypothesised that hyperglycaemia might provide an enriched environment for bacterial growth, shortening TTP. However it is possible that the glucose content in the culture medium already satifies bacterial growth requirements, negating the influence of hyperglycaemia [33]. Therefore the glucose levels in standard culture media are generally sufficient for bacterial growth, and additional glucose from hyperglycaemic conditions does not significantly influence TTP.

While our large population-based cohort is a key strength, this study has several limitations. The retrospective design precluded control over important variables such as the exact volume of blood inoculated into blood culture bottes, incubation conditions and the interval between sample collection and processing. These factors, along with the initial inoculum size, may have introduced variability into our TTP measurements. Additionally our reliance on ICD-10 coding for infection classification poses a risk of misclassification bias. Previous studies have validated the use of ICD-10 coding for comorbidities and infection site classification, accuracy varies depending on coding practices and condition specific factors [34]. This is particularly relevant for patients with multiple potential infection foci or an unclear primary focus, where misclassification may affect associations with TTP. However, despite these efforts, some degree of misclassification is inevitable, and this should be considered with interpreting the results.

Furthermore, we were not able to determine the time between culture draw and the time of initiation of incubation. Delays in transport may be an unadjusted confounding factor. Although these sources of variability were likely random, they may have influenced our results and determination of cut offs. Another important source of variability is the blood collection process itself. Blood draw difficulty and total volume collected can impact bacterial concentration in the sample, which may, in turn, influence TTP measurements [35]. Older patients often have more difficult venous access, potentially leading to lower collected blood volumes, while paediatric patients inherently have smaller blood volumes available for culture [35]. Given that lower blood volumes may result in shorter TTPs due to a higher bacterial concentration per millilitre of blood, this factor could partially explain the observed age-related variability in TTP. Future studies should consider accounting for blood volume variability o better assess its impact on TTP as a prognostic marker.

## Conclusion

This study identifies several novel associations influencing TTP, including shorter TTP linked to liver disease, malignancy, and specific bacterial isolates such as E. coli and Group A, B, C/G streptococci. Conversely, diabetes was not significantly associated with TTP, challenging prior assumptions. These findings highlight TTP’s potential as a marker of infection severity and prognosis. Further prospective studies are required to confirm its clinical relevance and establish its role in supporting personalised treatment strategies.

## Data Availability

Data cannot be shared publicly due to institutional ethics, privacy, and confidentiality regulations. Data release for the purposes of research under Sect. 280 of the Public Health Act 2005 requires application to the Director General (PHA@health.qld.gov.au).
